# Ulcerative and Spreading Nodular Lesion of Epizootic Lymphangitis in Adult Black Horse in Gondar, Ethiopia: Case Report and Wound Treatment

**DOI:** 10.1155/2024/2478774

**Published:** 2024-02-26

**Authors:** Balemual Abebaw

**Affiliations:** Department of Veterinary Clinical Medicine, Debre Tabor University, Debre Tabor, Ethiopia

## Abstract

An adult male black horse was presented to the UOG veterinary clinic with swelling, discharge, and skin wounds on the chest wall and the right front leg; then, it goes to the lateral and medial hind leg. Inspection and palpation were carried out, and we revealed that the skin was swelled, thickened and hard on clinical examination. There was also nodule rupture, and discharge on the limb and chest skin. Different therapy was applied starting from washing the wound with water-diluted potassium permanganate and then 0.9% NaCl together with paraffin topically placed on the wound. 10% povidone-iodine was also applied by using gauze. Griseofulvin 750 mg/day for one week was given as antifungal therapy orally. The prognosis was poor due to the long incubation period and high resistance of the causative agent.

## 1. Introduction

Epizootic lymphangitis is a chronic granulomatous contagious disease of horses, caused by Histoplasma farciminosum, characterized clinically by ulcerative, suppurative, spreading dermatitis, and lymphangitis [1–3]. It results from infection by a dimorphic fungus, Histoplasma capsulatum var. farciminosum [1, 4]. This organism has also been known as Histoplasma farciminosum. It is yeast-like cells with characteristic of double wall capsules, large ovoid or lemon-shaped refractile bodies, and replicate by budding [4, 5]. Epizootic lymphangitis mainly affects horses, donkeys, and mules. H. capsulatum var. farciminosum has also been reported in camels, cattle, and dogs [2, 6]. The disease is endemic in West and North Africa, East Africa (Ethiopia), and Asia, where it is mostly diffused in areas characterized by humid and hot climates [2, 7, 8]. Morbidity is high, and mortality is not high (10-15%), but the prolonged course and loss of function of affected animals can cause serious economic loss [9, 10]. The source of the organisms can be the skin lesions and nasal and ocular exudates of infected animals, or the soil. Fungal spores are transmitted from infected animals by direct contact or in animate objects such as bedding, grooming, utensils, or harnesses. Flies may also transmit the skin form mechanically when they feed on lesions and exudates [3]. The incubation period ranges from several weeks to six months [7, 9]. Following the initial invasion of the skin, the organism spreads through the lymphatic vessels to the regional lymph nodes and in more advanced cases involves the internal organs. Nodular and chronic suppurating lesions are evident in the skin overlying lymph vessels and nodes. Hematogenous spread (yeast cells are present intracellular or extracellular, especially of macrophages) with visceral involvement may occur which results in disturbance in general conditions of the animals [3, 7, 11]. The disease is characterized by a cord-like appearance of the subcutaneous lymphatic vessels, especially of the limbs, face, neck, and chest, and the development of a series of pyogranulomas, the discharge from which contains yeast-like cells of the pathogen. Rarely, infection may lead to pneumonia and conjunctivitis [1, 9]. The skin form of the disease may be confused with the skin form of glanders, ulcerative lymphangitis, sporotrichosis, and strangles [7]. The long incubation period of the disease, the high resistance of the causative agent, and the presence of clinically healthy carriers make control of the disease difficult in endemic areas [3, 4]. In most areas, epizootic lymphangitis has been eradicated by a strict policy of hygiene practices and culling or slaughter of infected animals. This case report describes that therapeutic response is poor, but control and prevention measures have the main role to protect against the transmission of the disease.

## 2. Case Report

### 2.1. Patient Information

An adult male black, white leg horse with an approximate weight of 300 kg was presented to the University of Gondar veterinary clinic with swelling, discharge, and skin wounds on the chest wall and the right front leg then goes to the lateral and medial hind leg. The case was started three weeks ago. There was no treatment or vaccination history before this time. The animal was free grazing on the pasture. Other horses were affected in the same case.

### 2.2. Clinical Findings

**Figure 1:**
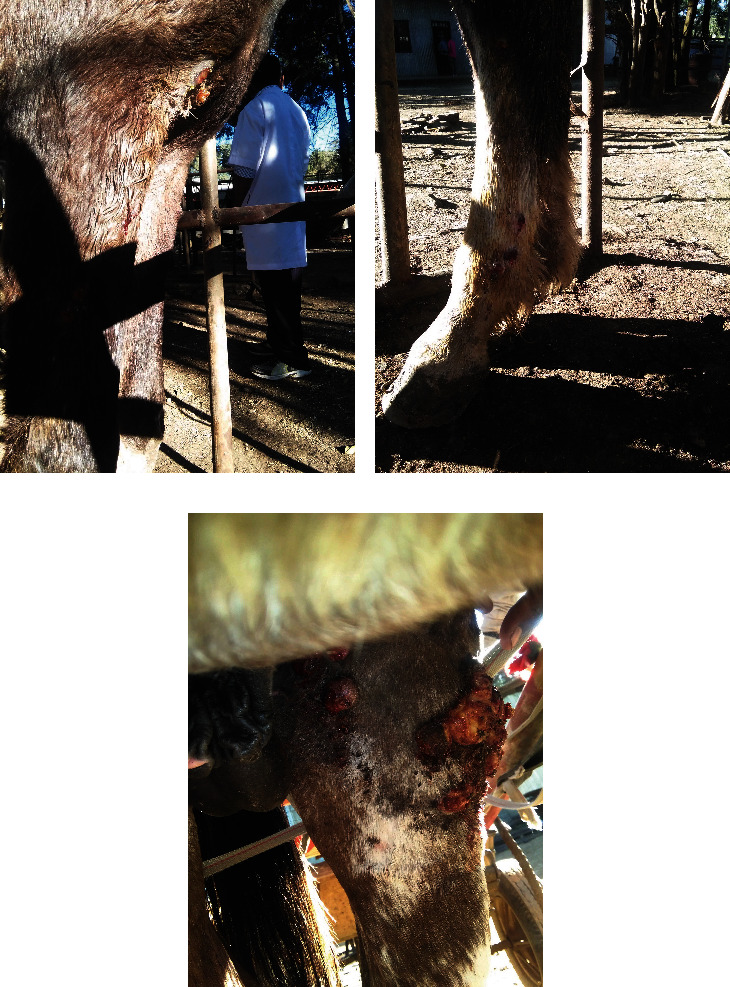
Clinical finding of epizootic lymphangitis in adult black and white horse: (a) lesion in the fore leg and chest, (b) lesion in the hind leg, and (c) nodules rupture and form skin lesion.

On presentation, the patient was bright, alert, and responsive with a normal mentation. Inspection and palpation were carried out, and we revealed that the skin was swelled, thickened, and hard on clinical examination. There was also nodule rupture and discharge on the limb and chest skin (Figure 1). The horse had a body temperature of 37.7°C, a heart rate of 38 beats per minute, and a respiratory rate of 12 breaths per minute.

### 2.3. Therapeutic Intervention

**Figure 2:**
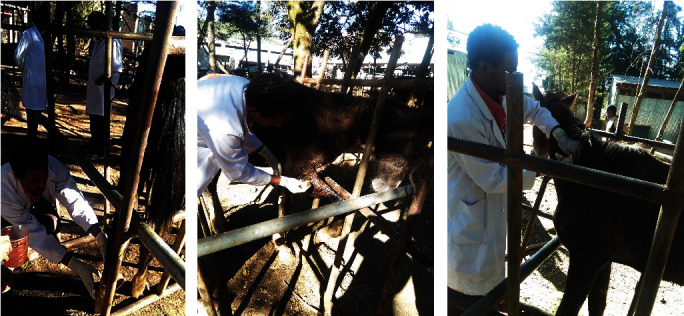
Treatment of epizootic lymphangitis: (a) the clinician has PPE washing the wound by water-diluted potassium permanganate, (b) 10% povidone-iodine is applied topically by using gauze, and (c) pen strip 12 ml IM for 3 days and griseofulvin 750 mg/day for one week orally were administered.

**Figure 3:**
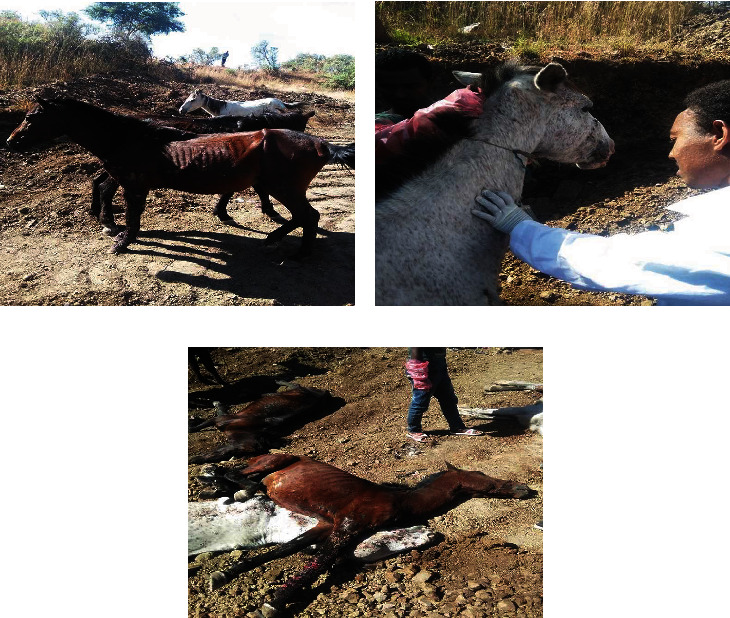
Control and prevention of epizootic lymphangitis: (a) epizootic lymphangitis affected horses near to UOG; (b) thiopental sodium and potassium chloride were administered in IV at the jugular vein at 20 ml and 60 ml, respectively; (c) finally, after the death of the affected horses, deep burrowing was carried out by tractor.

Different therapy was applied starting from washing the wound with water-diluted potassium permanganate and then 0.9% NaCl together with paraffin (as base for ointments) topically placed on the wound. 10% povidone-iodine was also applied by using gauze. For secondary complication, 12 ml pen strip (penicillin and streptomycin combination) IM for 3 days (at a dose of l ml/25 kg body weight) was administered. Griseofulvin 750 mg/day for one week was given as an antifungal orally for the treatment of epizootic lymphangitis (Figure 2). Even though different treatments were implemented, the prognosis was poor due to the long incubation period and high resistance of the causative agent. Prevention and control measures were carried out, and thiopental sodium 20 ml IV at the jugular vein (1 g powder by 20 ml water) and potassium chloride 60 ml IV at the jugular vein (30 g powder by 100 ml water) were used for euthanasia purposes for those horse that were affected by epizootic lymphangitis (Figure 3).

## 3. Discussion

Epizootic lymphangitis is a chronic disease, which causes pyogenic, ulcerative, and generalized spreading pyogranulomatous, multifocal dermatitis with lymphadenitis [7, 9, 10]. Many mildly affected horses might recover, and those that do might become immune for life, a belief that has led to a premium being placed in endemic areas on horses with characteristic scars [9, 12]. The disease is endemic in West and North Africa, East Africa (Ethiopia), and Asia, where it is mostly diffused in areas characterized by humid and hot climates [2, 7, 8]. In most areas, epizootic lymphangitis has been eradicated by a strict policy of hygiene practices, culling, or slaughter of infected animals [1, 2, 4] (Figure 3).

There are three forms of epizootic lymphangitis disease in horses: cutaneous, ocular, and respiratory. The cutaneous form is the most common, causing chronic, suppurative, ulcerating pyogranulomatous dermatitis, and lymphangitis [7, 13]. The skin form of the disease may be confused with the skin form of glanders, ulcerative lymphangitis, sporotrichosis, and strangles [7]. Strangles are caused by S. equi subsp. equi. The bacteria typically infect the URT and lymph nodes of the heads and necks (submandibular and retropharyngeal) [13, 14]. Glanders is caused by infection with the bacterium Burkholderia mallei. The pathogen causes nodules and ulcerations in the upper respiratory tract and lungs. Ulcerative lymphangitis is caused principally by Corynebacterium pseudotuberculosis. It is a lymphangitis of the lower limbs, marked by the presence of nodules and ulcers, which discharge green pus [13, 15]. Sporotrichosis is a noncontagious chronic infectious disease of equine. It is caused by Sporothrix shenki, which is a dimorphic fungus. It is characterized by cutaneous nodules and ulcers on the limb with or without lymphangitis and lymphadenitis [9, 14].

Different therapy was applied starting from washing the wound with water-diluted potassium permanganate. NaCl together with paraffin (a mineral oil that has been used as a laxative) was topically placed on the wound. 10% povidone-iodine was also applied by using gauze. For secondary complication, 12 ml pen strip (penicillin and streptomycin combination) IM for 3 days (at a dose of l ml/25 kg body weight) was administered. Griseofulvin 750 mg/day for one week was given as an antifungal for the treatment of epizootic lymphangitis. Even though different treatments were implemented, the prognosis was poor due to the long incubation period and high resistance of the causative agent (Figure 2).

Successful treatment with intravenous administration of sodium iodide, oral administration of potassium iodide, and surgical excision of lesions is limited since recurrences of clinical signs months later are possible [2, 15]. Sensitivity of the organism to amphotericin B, griseofulvin, nystatin, clotrimazole, and local medicinal plants has been reported. However, in most areas, epizootic lymphangitis is a notifiable disease, and treatment is not allowed, and thereby, diseased animals must be forsaking [8, 16, 17]. Prevention and control measures were carried out, and thiopental sodium 20 ml IV at the jugular vein and potassium chloride 60 ml IV at the jugular vein were used for euthanasia purposes for that horse that was affected by epizootic lymphangitis. Finally, after the death of the affected horses, deep burrowing was carried out by tractor to prevent dissemination of the fungus to the other horses (Figure 3).

## Data Availability

The material set used in this review is available from the corresponding author and can be accessed through reasonable request.
